# Intrinsic Axonal Growth and the Drive for Regeneration

**DOI:** 10.3389/fnins.2016.00486

**Published:** 2016-10-27

**Authors:** Kevin J. O'Donovan

**Affiliations:** Department of Chemistry and Life Science, United States Military AcademyWest Point, NY, USA

**Keywords:** axon growth, neuronal regeneration, intrinsic growth, mouse models, neuronal injury

## Abstract

Following damage to the adult nervous system in conditions like stroke, spinal cord injury, or traumatic brain injury, many neurons die and most of the remaining spared neurons fail to regenerate. Injured neurons fail to regrow both because of the inhibitory milieu in which they reside as well as a loss of the intrinsic growth capacity of the neurons. If we are to develop effective therapeutic interventions that promote functional recovery for the devastating injuries described above, we must not only better understand the molecular mechanisms of developmental axonal growth in hopes of re-activating these pathways in the adult, but at the same time be aware that re-activation of adult axonal growth may proceed via distinct mechanisms. With this knowledge in hand, promoting adult regeneration of central nervous system neurons can become a more tractable and realistic therapeutic endeavor.

The focus of this review is a description of the mechanisms of intrinsic axonal growth during neuronal regeneration. Before describing a sampling of classical and recent work in regeneration, I provide a short review of developmental axon growth.

The precise execution of axon growth is essential for the proper wiring of the developing nervous system. Molecular and genetic analyses over the past few decades have identified many of the pathways and molecules that orchestrate the intricate processes of correctly guiding and growing axons over what can be very long distances during mammalian development. As an example, some dorsal root ganglion (DRG) axons grow to well over 1 meter in humans with the axon spanning from the tip of a limb to as far up as the brainstem (reviewed in Goldberg, 2003). This molecular interplay of extracellular growth and guidance factors with plasma membrane-bound receptors and their intracellular signaling pathways drives the process of axonal growth throughout the nervous system during embryonic and postnatal development. Once the nervous system has been wired and becomes functional, the intrinsic growth capacity of most neurons becomes greatly diminished (Chen et al., 1995; Bandtlow and Löschinger, 1997; Fawcett, 1997; Goldberg, 2003). This makes intuitive sense since in a normally functioning adult animal, long distance growth would typically no longer be required. Instead, as has been described previously, stabilization of neuronal circuits would be favored (Purves et al., 1988; O'Leary et al., 1994). This review will focus on the pioneering developmental studies that defined the factors and pathways critical for long distance axonal growth and how those studies informed subsequent, more recent studies that attempt to re-activate the intrinsic growth capacities of neurons in the context of central nervous system regeneration.

## Developmental axon growth

### Neurotrophins

The neurotrophins (NGF, BDNF, NT-3, and NT-4/5) and their cognate receptors [TrkA (*Ntrk1*), TrkB (*Ntrk2*), and TrkC (*Ntrk3*)] regulate neuronal survival, growth, and differentiation throughout the nervous system (Reichardt, 2006). Early studies revealed that many subclasses of sensory and sympathetic neurons, whose axons must grow long distances in order to innervate peripheral target fields, were dependent on the various neurotrophins (Table 1). Mice lacking nerve growth factor (NGF) (Crowley et al., 1994) or its receptor TrkA (Smeyne et al., 1994; Silos-Santiago et al., 1995) fail to develop nociceptive sensory and sympathetic neurons. BDNF- and TrkB-deficient mice (Klein et al., 1993; Ernfors et al., 1994a) also showed sensory defects, in particular in certain central nervous system (CNS) neurons as well as the vestibular, trigeminal, and dorsal root ganglia. Similarly, disruption of either NT-3 (Ernfors et al., 1994b) or its receptor TrkC (Klein et al., 1994) caused defects in DRG proprioceptive sensory neurons. Both NT-3- and TrkC-null mice exhibited a lack of proprioceptive type Ia muscle afferents that project to spinal motor neurons and defects were also observed for large myelinated axons in the dorsal root and dorsal columns as well as in muscle spindles. Because neurotrophins were required for the survival of neurons whose axons would normally grow their axons over long trajectories, it was difficult to simultaneously determine the extent to which neurotrophins could also regulate axon growth. This was the case until Korsemeyer and colleagues discovered that the pro-apoptotic protein Bax was required for the death of sensory neurons undergoing NGF deprivation (Deckwerth et al., 1996), and that the surviving neurons would subsequently respond to a re-application of neurotrophin by extending more neurites. This observation made it feasible, in the context of Bax-deficient mice, to ask survival-independent questions about neurotrophin function. Indeed, these studies revealed that Bax null sensory neurons cultured from embryonic (E) days 11–13 were unipolar with only a short process and that upon addition of neurotrophins, the cells elaborated a second axon and robust lengthening of both axons (Lentz et al., 1999). While NT-3 induced marked terminal branching of some axonal processes, NGF instead induced axon elongation of a different subtype of neurons. Subsequent studies (Patel et al., 2000) in which the survival of either NGF- or TrkA-deficient DRG sensory neurons was rescued by the concomitant removal of Bax revealed that the peripheral projections of NGF- or TrkA-deficient nociceptive neurons failed to grow into their target fields in the skin. Survival of the neurons alone was not enough to get the neurons to proper location. Interestingly, however, the NGF- or TrkA-deficient central projections did target correctly to the dorsal horn of the spinal cord, though they failed to undergo proper differentiation (Patel et al., 2000). These experiments in which the neurotrophins were defined as critical regulators of axonal growth set the stage for a molecular dissection of the pathways downstream of the Trk receptors.

**Table 1:** **Partial summary of how the neurotrophins, RAF/MEK/ERK, PI3K/Akt/mTOR, and JAK/STAT pathways are involved in development and regeneration**.

	**Development**			**Regeneration**		
	**Effector**	**Function**	**System**	**Effector**	**Function**	**System**
Neurotrophins	TrkA/NGF	survival, axon growth	nociceptive DRG, sympathetic	NT-3	axon growth	spinal cord
	TrkB/BDNF	survival, axon growth	DRG, central sensory	TrkB	axon growth	corticospinal tract
	TrkC/NT-3	survival, axon growth	proprioceptive DRG			
RAF/MEK/ERK	B-Raf	axon growth	nociceptive DRG	B-Raf	axon growth	dorsal root entry zone, optic nerve
	SRF	axon branching	nociceptive DRG	ERK	axon growth	DRG
	CREB	survival, axon growth	sensory, sympathetic	NF-1	axon growth, branching	dorsal root entry zone
	Egr3	survival, axon growth	muscle spindle, sympathetic	DLK	axon growth	DRG
	ETS family	axon growth, branching	proprioceptive, motor			
PI3K/Akt/mTOR	PTEN	axon branching	RGC tectum	mTOR	axon growth	corticospinal, optic nerve, spinal cord
				PTEN/Akt	dendrite growth	*Drosophila* motor neuron
JAK/STAT	gp130	axon growth	sympathetic	SOCS3	axon growth	corticospinal, optic nerve, spinal cord
				JAK/STAT	axon growth	DRG conditioning lesion
				IL-6	axon growth	DRG

*Dorsal root ganglion (DRG), retinal ganglion cells (RGC)*.

### Downstream effectors of the neurotrophins and their role in axon growth

Neurotrophins signal via the Trks, which are single pass transmembrane tyrosine kinase receptors. In turn the Trks signal to several downstream pathways including the MAP kinase (RAF-MEK-ERK), PI3K-Akt-mTOR, and PLC-γ pathways. Canonical signaling downstream of the Trks proceeds via Ras which mediates signaling to both Raf and PI3K. Raf is categorized as a MAP kinase kinase kinase or MAP3K indicating it lies upstream of subsequent downstream kinases Mek, a MAP2K, and Erk, a MAPK. PI3K or phosphoinositide-3-kinase generates phosphatidylinositol trisphosphates which serve to activate Akt and further downstream mTOR.

Pioneering work (Vogel et al., 1995) from the Parada lab showed that loss of the neurofibromatosis gene NF1, a repressor of Ras, endowed embryonic DRG, trigeminal and sympathetic neurons the ability to grow and extend neurites in the absence of neurotrophins, thus placing Ras proximal to the neurotrophin receptors in terms of both survival and axonal growth. Subsequently, in experiments designed to dissect which pathways downstream of the Trks were required for axon growth, the Snider lab showed in embryonic DRG Bax null neurons that a dominant negative blockade of either the PI3K or MAP kinase pathways alone or both together could inhibit NGF-induced axon growth (Markus et al., 2002). In the same model system, they asked what was sufficient to drive axon growth in the absence of NGF by transfecting membrane-targeted constructs of Akt, PI3K or Raf to render them constitutively active. While constitutively active RAF drove long distance axon growth, both Akt and PI3K activation increased axon caliber while active Akt promoted axon branching. This finding was reminiscent of previous observations that NGF promoted long distance growth while NT-3 drove terminal branching (Lentz et al., 1999) and indeed Markus et al. found that NT-3, when compared to NGF, caused preferential activation of Akt. Together, these data demonstrated that both the PI3K and MAP kinase pathways were required for NGF-induced axon outgrowth in embryonic DRG neurons, and that either of the pathways were also sufficient to drive growth though the growth was qualitatively different depending on the pathway. Later studies from the Holt lab support the observation that the PI3K pathway promotes axon branching as they show that PTEN, a negative regulator of the PI3K pathway, is regulated by the E3 ubiquitin ligase Nedd4 and that loss of PTEN promotes branching of retinal ganglion cells (RGC) in the *Xenopus* tectum (Drinjakovic et al., 2010).

In analogous experiments with RGC's (Matter and Mellman, 1994), the authors over-expressed the pro-survival protein Bcl-2 in order to determine the survival-independent growth requirements for these cells. While all the neurotrophins and several other growth factors were capable of driving axon growth, BDNF, CNTF, and IGF-1 had the strongest growth-promoting effects. Further studies by Barres and colleagues revealed that pharmacologic inhibition of the PI3K and MAP kinase pathways had to be combined in order to completely inhibit axon growth, while blockade of either pathway alone led to a partial inhibition. In sympathetic neurons, Kaplan, Miller and colleagues mutated the BDNF receptor TrkB in order to identify the relevant downstream pathways that are required for survival and axon growth (Atwal et al., 2000). While TrkB mutants that abrogated PLC-γ signaling had no significant effect on survival or growth, TrkB mutated at its Shc site, which abrogates signaling to both the PI3K and MAP kinase pathways, caused a severe impairment in axon growth and neuron survival. Further analysis suggested that neuron survival was more dependent on the PI3K pathway than the MAP kinase pathway while robust axon growth required both pathways.

### Transcription factors and developmental axon growth

Several transcription factors have been shown to be involved in axon growth during development. Some of these transcription factors are downstream of the neurotrophins and help orchestrate the axonal growth program. In particular, CREB, which gets phosphorylated on its serine 133 in a neurotrophin-dependent manner, was shown to be required for the survival and axon growth of both sensory and sympathetic neurons (Lonze et al., 2002). Conditional SRF null mice in which serum response factor SRF is absent in dorsal root ganglia neurons show defects in NGF-dependent axonal terminal branching in the limbs (Wickramasinghe et al., 2008), while *in vitro* studies demonstrate that SRF is both necessary and sufficient for NGF-mediated axon growth.

The ETS transcription factor family also plays a role in axonal growth of both sensory and motor neurons. Er81 (*Etv1*) is required for the proper growth of group Ia proprioceptive neurons that project onto ventral spinal cord motor neurons (Arber et al., 2000) and a similar phenotype was observed in NT-3 null mice that had been crossed onto the Bax null background (Patel et al., 2003), Notably, these NT-3^−/−^: Bax^−/−^ lacked Er81 expression in their proprioceptive neurons. Somewhat reminiscent of the SRF knockout defects in skin innervation, certain motor neurons axons in PEA3 (*Etv4*) null mice fail to branch properly in the target muscles (Livet et al., 2002).

The zinc finger-containing immediate early gene transcription factor Egr3 has a demonstrated role in axon growth (Eldredge et al., 2008; Quach et al., 2013). Interestingly, Egr3 null mice have degenerating muscle spindles, (Tourtellotte and Milbrandt, 1998) which fail to express NT-3, which results in type Ia afferents failing to reach their targets in the muscle spindle (Chen et al., 2002). Also, Egr3 is required for sympathetic axons to properly innervate their targets (Eldredge et al., 2008) and these defects appear to be dependent on growth, but not survival since Egr3 null mice crossed onto a Bax null background fail to innervate several sympathetic nervous system targets including the heart, spleen, kidney and pineal gland (Li et al., 2011). Egr3 is also required for proper innervation of salivary glands and heart where NGF and NT-3 expression persist in the absence of Egr3 suggesting that Egr3 regulates axon growth via neurotrophin-independent factors in these contexts. More recent studies have demonstrated a role for Egr3 in sympathetic neuron axon branching and dendritic morphology (Quach et al., 2013).

The Brn3a (*Pou4f1*) transcription factor is expressed in several sensory neuronal populations including the DRG and trigeminal ganglia. Brn3a knockout mice exhibit sensory neuron growth defects wherein two major branches of the trigeminal nerve, the maxillary and occipital, exhibit excessive branching not observed in wild type mice suggesting Brn3a may regulate a repulsive factor (Eng et al., 2001). Also, Brn3a null mice do not properly innervate the whisker follicles. Importantly the growth defects observed in these mice occur prior to sensory neuron death demonstrating that Brn3a's effects on growth are likely separable from its effects on survival. Further analyses revealed that these Brn3a defects cannot be accounted for by changes in Trk receptors, since TrkA and TrkB expression levels go down later in these animals while TrkC null embryos do not exhibit the same abnormal branching seen in the Brn3a mice. Neuropilin (*Npn1*) expression levels likewise do not change in these mice and so is likely not the cause of the defect.

The runt domain transcription factors Runx1 and Runx3 are involved in the fate specification and axonal targeting of cutaneous (Chen et al., 2006; Marmigère et al., 2006) and proprioceptive (Kramer et al., 2006) sensory neurons, respectively and that Runx1 is sufficient to drive axonal growth (Marmigère et al., 2006).

Regarding the Kruppel-like factors (KLF) family of transcription factors, the Goldberg lab screened for genes that affected the intrinsic outgrowth (Moore et al., 2009) of RGC's and found that several members of the KLF family were either positive and negative regulators of this process. The positive regulators of RGC growth tended to be developmentally down-regulated while the opposite was the case for the negative regulators of axonal growth. These observations were consistent with prior observations that RGC lost their intrinsic growth capabilities during development (Goldberg et al., 2002).

In mice lacking 3 NFAT genes (NFATc2, NFATc3, and NFATc4) or calcineurin signaling, sensory neurons failed to grow axons *in vitro* in response to neurotrophins (Graef et al., 2003). Commissural axons from these mice also failed to respond to netrin, suggesting that the NFAT family may mediate neuronal growth signals in different neuronal populations.

### gp130 receptors and the downstream JAK/STAT pathway

As reviewed previously (Zigmond, 2012), the gp130 cytokine receptors are required *in vivo* for what is often referred to as the cholinergic switch of superior cervical ganglion (SCG) sympathetic neurons from an early noradrenergic phenotype to a mature cholinergic phenotype upon innervation of sweat glands (Schotzinger and Landis, 1988, 1990; Stanke et al., 2006). The gp130 receptors serve as a receptor for a variety of cytokines including IL-6, CNTF, and LIF which signal via their downstream partners the JAK tyrosine kinases and STAT transcription factors. The pathway can be negatively regulated by a STAT inhibitor known as suppressor of cytokine signaling (SOCS3), which binds to and represses both JAK and gp130 receptors (Chen et al., 2000).

### Regeneration, a re-activation of intrinsic growth pathways

The data that certain factors or pathways are important for developmental long distance axonal growth sets the stage for what may be possible during adult axon regeneration. These data should likewise serve notice that this process is inherently multi-factorial and necessarily complex. The observation that damage to the spinal cord could result in profound and often permanent motor and sensory loss is an ancient one. The Edwin Smith Papyrus, a medical treatise that dates back 5000 years, describes in detail several cases involving spinal cord injury (van Middendorp et al., 2010), where some are treatable and others are not. In the modern era, Ramon y Cajal recognized and experimented on the same problems facing the field today (Ramón and May, 1928). Indeed, he challenged future scientists when he wrote “*Pathologists consider it an unimpeachable dogma that there is no regeneration of the central paths, but we and others have demonstrated beyond doubt that there is a production of new fibers and clubs of growth in the spinal cord following different types of insults…It is for the science of the future to…work to impede or moderate the gradual decay of neurones, to overcome the almost invincible rigidity of their connections, and to establish normal nerve paths, when disease has severed centers that were intimately associated*” (Lobato, 2008).

The classic experiments of Aguayo, Bray and colleagues showed that central neurons indeed have the capacity to re-grow if provided with a permissive environment, in this case a sciatic nerve graft (Aguayo et al., 1981; David and Aguayo, 1981; Richardson et al., 1984). Their seminal observations caused a paradigm shift in the field of regeneration, and to this day more than 30 years after their original observation, the field is still asking detailed questions that stem from their work. Specifically, using a variety of techniques, scientists have been testing whether a removal of inhibitory signals can promote axon growth (reviewed in Lee and Zheng, 2012; Pernet and Schwab, 2012). This field has turned out to be much more complex than initially appreciated, especially given the observations that Nogo and its cognate receptors (NgRs), molecules which have previously been shown to be inhibitory to axonal growth, can stabilize and restrict synapse formation in the developing CNS (Pradhan et al., 2010; Zagrebelsky et al., 2010; Wills et al., 2012).

In contrast to attempts to promote neuronal growth by removal of inhibition, researchers have more recently asked whether the relative intrinsic growth capacity of adult or injured neurons may have some impact on growth and whether the intrinsic growth properties of adult neurons can be re-activated to drive regeneration. Barres, He and others have shown that during development (Goldberg et al., 2002; Liu et al., 2010) or after injury (Park et al., 2008) that neurons lose some of their intrinsic growth capacity. Importantly, however, some adult neurons retain their capacity to regenerate and re-form synaptic connections. Therefore, the presumption is that their intrinsic growth remains intact or can be re-activated.

## Re-activation of intrinsic growth

### The conditioning lesion

Since the pivotal observation (McQuarrie and Grafstein, 1973) that an initial lesion of adult peripheral nerve could elicit enhanced axonal growth capacity of these same neurons following a second lesion, researchers have extensively studied this remarkable phenomenon to better understand and develop strategies to promote axonal regeneration of injured neurons. Some of these earlier studies (Richardson and Issa, 1984) showed that the peripheral lesion could potently induce regrowth of centrally projecting sensory axons into peripheral nerve grafts. Mechanistic insight into the conditioning lesion-induced intrinsic growth was provided by the Skene lab, (Smith and Skene, 1997) which found that new transcription was required post-injury in order to get long distance growth. They further showed that the lesion-induced growth could be recapitulated in part by inhibition of retrograde transport *in vivo*. Subsequently, it was demonstrated that a conditioning lesion could promote growth of the central projections into the spinal cord itself (Chong et al., 1999) or when applied 1–2 weeks prior to a dorsal column injury, the conditioning lesion could induce axonal regeneration into and above the lesion site (Neumann and Woolf, 1999). The conditioning lesion also is effective in driving sympathetic axons to regrow when the neurons are subsequently grown in culture (Shoemaker et al., 2005). Interestingly, a conditioning lesion in *Drosophila* motor neurons protected them from axonal degeneration (Xiong and Collins, 2012), though it did not promote axonal growth in this context. While more recent studies have taken advantage of transgenic or knockout mice to interrogate the molecular pathways that underlie the conditioning lesion, an important early observation was made in the PNS where the Snider lab showed that phospho-STAT3 was robustly induced in the nuclei of injured DRG neurons (Liu and Snider, 2001). Further, using a pharmacological approach they showed that when added to DRG cultures from treated animals, a JAK/STAT inhibitor, but not MEK or PI3K inhibitors, could block the conditioning lesion-induced axon growth.

Indeed, a series of papers showed that spinal axon regeneration following a conditioning lesion requires LIF (Cafferty et al., 2001), IL-6 (Cafferty et al., 2004), and intact JAK/STAT signaling (Qiu et al., 2005) where phospho-STAT3 was induced in DRG neurons. While there is still some disagreement (Richter et al., 1990) about the role IL-6 plays in this process, the observation that cytokines such as LIF and IL-6 as well as downstream signaling molecules were required for conditioning lesion-induced regeneration coupled with the prior observation (Zhong et al., 1999) that IL-6 was likewise required for the proper regeneration of peripheral sensory axons provided strong evidence that the immune system played a key role in the regenerative response. More recently, intrathecal delivery of IL-6 for 1 week was found induce expression of mTOR, promote axonal sprouting adjacent to the injury site, and, remarkably, significant functional recovery in a model of corticospinal tract (CST) injury (Yang et al., 2012).

Another notable study (Blesch et al., 2012) used a computational approach to globally compare the differences between a conditioning lesion and cAMP treatment in the context of central axon regeneration and found, perhaps not too surprisingly, that the conditioning lesion elicited a much broader set of genetic pathways that cAMP alone. Indeed, this result may be indicative of a theme for successful regeneration in many model systems. That is, for robust regeneration to occur, multiple pathways must be activated either by a conditioning lesion or via a genetic manipulation (loss of PTEN) that is sufficiently upstream. Consistent with this notion is the fact that combinatorial treatment regimens have proved to be beneficial for regeneration (Kadoya et al., 2009; Kurimoto et al., 2010; Sun et al., 2011).

Interestingly, recent work from the Zou lab demonstrated a novel ethidium bromide-induced conditioning lesion (Hollis et al., 2015). Here, when injected into the sciatic nerve, ethidium bromide acted as a demyelinating agent and induced prototypical regeneration associated genes as well as robust central regeneration that surpassed that induced by traditional nerve crush. Further, they showed that while no macrophages were induced in the periphery, there was evidence for macrophage infiltration in the DRG.

### Re-activation of the Akt-mTOR pathway

Several papers by the He lab have demonstrated the power of de-repressing an otherwise dormant growth pathway in adult mouse neurons (Table 1). First, in a model of optic nerve crush, the He lab showed that loss of PTEN, as well as loss of TSC1, both repressors of the mTOR pathway, caused robust and long distance regenerative growth of the injured RGC neurons (Park et al., 2008). In subsequent studies in the optic nerve crush model, they demonstrated sustained regenerative synergy when loss of PTEN was combined with loss of suppressor of cytokine signaling 3 (SOCS3), thereby leading to activation of both the mTOR and JAK/STAT pathways (Sun et al., 2011). Previously, it had been shown that removal of SOCS3 alone was sufficient to promote optic nerve regeneration and that this effect depended on the gp130 receptor (Smith et al., 2009).

Recent work (Li et al., 2015) from the Liu lab has shown that following a pre-chiasm lesion, simultaneous selective deletion of mTOR and SOCS3 promotes a functional regeneration of mouse RGC neurons to the suprachiasmatic nucleus. The authors further show that while the innervation pattern and electrophysiological responses are abnormal, functional synapses were formed. Interestingly, work from a collaboration of the He and Sanes labs has demonstrated that alpha RGC's, when compared to other RGC subtypes, preferentially survive axotomy and exhibit robust regeneration (Duan et al., 2015). Of note, alpha RGC's express high mTOR levels. That said, a more global proteomic analysis of injured RGC's (Belin et al., 2015) revealed that in addition to decreased mTOR levels, these cells also show down-regulation of c-myc protein. Notably here, the authors made a non-specific preparation of RGCs and did not preferentially purify alpha RGCs during their extractions. That said, one would predict alpha RGC's would express high levels of c-myc in addition to mTOR. The authors go on to show that forced c-myc expression in RGC's drives regeneration and that mTOR and c-myc, both of which are potent growth-promoting genes, are not down-regulated in injured peripheral neurons, and thus may be required in some capacity for proper peripheral regeneration.

**Figure 1:**
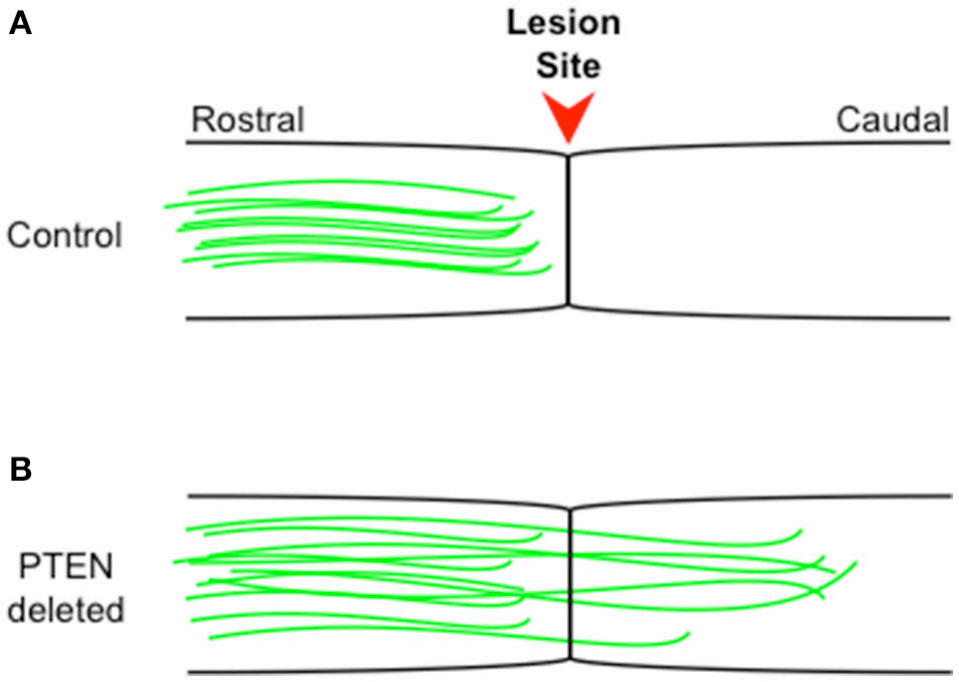
**Corticospinal tract regeneration proceeds in the absence of PTEN**. Schematic representation of sagittal spinal cord sections from control wild type **(A)** and PTEN deleted **(B)** mice. Intact corticospinal tract neurons (green) rarely regenerate beyond the injury site (arrowhead) into more caudal regions, while in PTEN deleted **(B)** mice, intact corticospinal tract neurons (green) consistently regenerate well beyond the injury site into more caudal regions even 1 year following the initial injury. The figure is based on primary data presented previously (Liu et al., 2010; Du et al., 2015).

Further studies have shown that PTEN loss can also drive regeneration in the adult CST (Liu et al., 2010), an environment much less conducive to regeneration than that of the optic nerve. Following a spinal cord lesion, animals in which PTEN has been conditionally deleted showed robust axonal regeneration into and past the lesion site and making synaptic connections distal to the lesion (Figure 1). Recently, the He lab has shown (Jin et al., 2015) using a pyramidotomy model that concomitant deletion of SOCS3 and PTEN leads to sprouting of intact corticospinal tract neurons and, impressively, these animals showed improved performance on a horizontal ladder test when compared to control animals. Even 1 year after spinal cord injury PTEN deletion is effective at promoting CST regeneration beyond the lesion site (Du et al., 2015).

#### mTOR and neural stem cell transplants

Work from the Tuszynski lab has shown in rats that following a T3 complete transection of the spinal cord, transplantation of stem cells leads to long distance axon growth that traverses the lesion site and traveling extensively both rostrally and caudally in the spinal cord (Lu et al., 2012). For the grafting experiments, either neural stem cells derived from embryonic rats or human stem cells lines were mixed with a fibrin matrix containing 9 growth factors including BDNF, NT-3, HGF, IGF-1, and then transplanted into the injury site. The growth factors support the survival of the grafted stem cells but also likely drive axonal growth of the neural stem cells. Consistent with this observation, the authors also show that systemic administration of the mTOR inhibitor rapamycin significantly inhibited axonal growth following graft transplantation, suggesting that sustained mTOR signaling in the neural stem cells is required for long distance growth as was demonstrated previously by the He lab in models of optic nerve and CST regeneration (Park et al., 2008; Liu et al., 2010; Sun et al., 2011). Rats that received the transplant exhibited improved functional recovery in an open field locomotion study with a maximal BBB score (Basso et al., 1996) of 7 at 4–6 weeks post-graft. The authors go on to show that the recovered rats exhibited an intact electrophysiological signal when stimulation took place rostral to the injury while recordings were made caudal to the transection. Rats that were transected but that did not receive a graft had no electrophysiological transmission.

#### mTOR and fly regeneration

Work from the Jan labs has shown that the mTOR pathway is also important for regeneration in fly. Their paper is notable for several reasons. First, they have established a novel model system in fly in which one can analyze both peripheral and central nervous system axon regeneration as well as the little studied phenomenon of dendritic regeneration (Song et al., 2012). Their model makes use of a subclass of mammalian DRG-like dendritic arborization (da) neurons, whose peripherally located cell body extends dendrites to the body wall and an elaborate axon that traverses several tissues before entering and forming synaptic connections in the ventral nerve cord, which is part of the CNS in *Drosophila*. Amazingly, as in mammals, these da neurons regenerate in the periphery but fail to do so upon entering the CNS. Further, consistent with what the He lab has shown (see above), either loss of PTEN or Akt activation promoted robust cell-autonomous regeneration beyond the lesion site and into the target area. They go on to demonstrate that da neuron dendrites likewise regenerate and that the process is developmentally regulated. Further, the PTEN-Akt pathway regulates the observed regeneration in both extrinsic (non-cell-autonomous) and intrinsic (cell-autonomous) manners. Intriguingly, the extrinsic regulation of da dendrite regeneration is under the control of the miRNA *bantam*, which is expressed in adjacent epithelial cells and had previously been shown to regulate da dendrite growth via Akt inhibition. (Parrish et al., 2009). Manipulations of *bantam* had no effect on da axon regeneration.

### The MAP kinase pathway in regeneration

Following sciatic nerve injury Fainzilber and colleagues showed (Perlson et al., 2005) that Erk1 and Erk2 are phosphorylated in axons and concomitantly bind the intermediate filament protein vimentin. Inhibition of the Erk-vimentin interaction, via pharmacologic means or in vimentin null mice impaired DRG regeneration. In 2009, Tuszynski and colleagues showed that combination of both intrinsic and extrinsic factors could promote axonal regeneration or bridging across spinal injuries (Kadoya et al., 2009). The successful combination included conditioning lesion, transplantation of bone marrow stromal cells and a neurotrophin-3 (NT-3) gradient, while partial treatments failed to promote effective bridging.

Adult corticospinal tract neurons fail to regenerate following injury suggesting that their intrinsic growth capacities have diminished in the adult. To test whether these descending motor neurons could be re-activated, the Tuszynski lab transduced CST layer V neurons with a lentivirus expressing the BDNF receptor TrkB and following injury in intervening areas, the CST neurons were able to regenerate into spinal regions expressing BDNF. If TrkB was mutated to abolish downstream ERK signaling, then regeneration was impaired. (Hollis et al., 2009).

Consistent with a positive role for the MAP kinase family in regeneration, as well as PI3K/Akt pathways in axonal growth and building on previously described work (Vogel et al., 1995) from the Parada lab, loss of neuronal NF1 leads to enhanced sensory neuron axonal outgrowth and branching as well as functional recovery following a dorsal root lesion in mice (Romero et al., 1998).

In mammals, dual leucine zipper kinase or DLK1 (MAP3K12) knockout and gene-trap mice exhibit impaired peripheral axon regeneration (Itoh et al., 2009; Shin et al., 2012). In the complete absence of DLK1 (Shin et al., 2012), neither phospho-STAT3 (pSTAT3) or phospho-c-jun were up-regulated in the cell body following injury, while pSTAT3 was induced in axons but did not undergo retrograde transport in DLK1 null mice. Complementary results were obtained in the DLK1 gene trap mice in which phospho-c-jun levels were reduced following a sciatic nerve axotomy as well as reduced neurite outgrowth from DRG explants. These effects were seemingly in contrast to findings showing the DLK drives apoptosis of axons (Miller et al., 2009; Ghosh et al., 2011; Itoh et al., 2011). One model (Shin et al., 2012; Tedeschi and Bradke, 2013) suggests that DLK drives regeneration via retrograde transport in the proximal axon while promoting degeneration in the distal axon.

**Figure 2:**
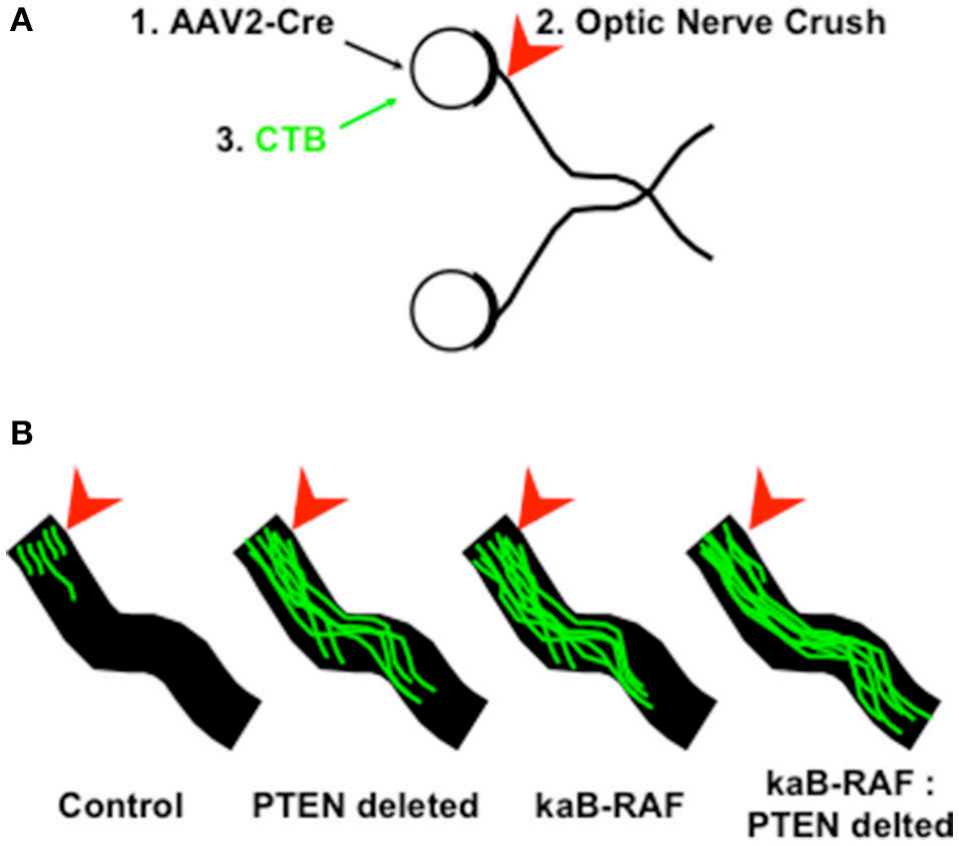
**MAP kinase pathway activation and de-repression of the PI3K/Akt pathway synergizes optic nerve regeneration. (A)** Optic nerve crush experiments proceeded as follows: (1) Cre-expressing Adeno-associated virus-2 (AAV2) was injected into the eye 2 weeks prior to injury to allow for Cre expression to build up in retinal ganglion cells which are preferentially transduced by AAV2; (2) Optic nerves are crushed (arrowhead) in control and AAV2-Cre transduced animals and (3) Cholera Toxin B (CTB) conjugated to Alexa Fluor 488 is injected into the eye and tissue was processed for optic nerve imaging. **(B)** Representative images of regenerating optic nerves (green) from indicated genotypes: Control, PTEN deleted, kinase-active B-RAF (kaB-RAF), and the combination of both PTEN deletion and kaB-RAF. The figure is based on data presented previously (O'Donovan et al., 2014).

**Figure 3:**
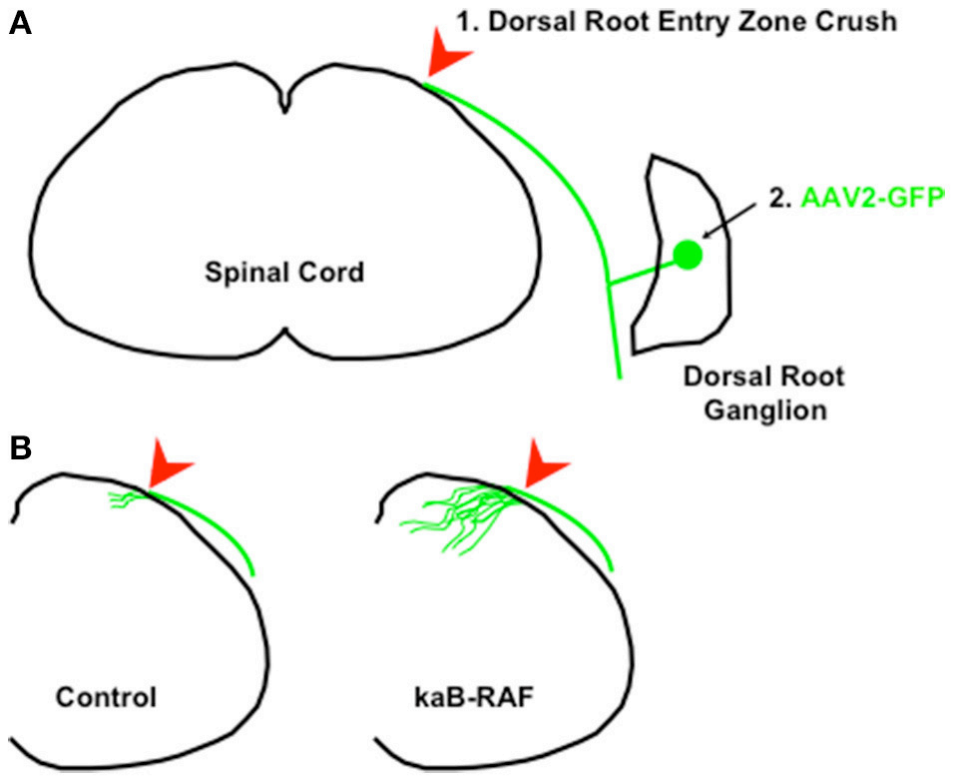
**Constitutive B-RAF drives dorsal root entry zone regeneration. (A)** 1. The dorsal root is crushed at the dorsal root entry zone. 2. To visualize dorsal root ganglion neurons, AAV2-GFP is injected into either adult control mice or adult kaB-RAF-expressing mice. **(B)** Two weeks following crush, the processed tissue reveals robust regeneration into the spinal cord in B-RAF-expressing mice (right) but not in control mice (left). The figure is based on data presented previously (O'Donovan et al., 2014).

More recently, we wanted to know what was sufficient to drive DRG axon growth *in vivo*. To do so, we used a Cre-inducible constitutively active or kinase-active B-RAF (kaB-RAF) knock-in to drive the RAF-MEK-ERK pathway in embryonic DRG's *in vivo*. These experiments showed in a TrkA null background, thus in the absence of upstream neurotrophin signaling for nociceptive neurons, that kaB-RAF is sufficient to drive robust developmental axon growth of DRG neurons (O'Donovan et al., 2014). We further showed in both adult optic nerve (Figure 2) and dorsal root injury (Figure 3) models that kaB-RAF induced regenerative axon growth and that the combination of kaB-RAF and PTEN loss was synergistic in injured optic nerve.

### Transcription factors in regeneration

Work from the Goldberg lab identified the Kruppel like transcription factor 4 in a screen for developmentally regulated RGC genes that could modulate neuronal growth. Indeed, subsequent *in vivo* studies showed that knockout of KLF4 caused an increase in growth over controls following optic nerve injury of RGCs. Thus, KLF4 appeared to be actively repressing growth in the adult (Moore et al., 2009). They went on to characterize other KLF members, which exhibit either pro or anti-growth activities. One factor, KLF7, was shown to drive growth when transfected into cortical neurons. Interestingly, while it had been previously shown that both zebrafish RGCs and mammalian DRGs up-regulate KLF7 following injury (Veldman et al., 2007), there was no such induction in the spinal cord following a CST transection (Blackmore et al., 2012). However, when an N-terminal truncation of KLF7, optimized to enhance expression and growth promotion, is fused to the heterologous VP16 transactivation domain that it can promote sprouting and regeneration following a CST lesion (Blackmore et al., 2012).

Work from the Woolf lab has identified the ATF3 transcription factor, a member of the ATF/CREB family of transcription factors that bind to the CRE binding sequences, on the basis of its induction in all DRG neurons following a peripheral, but not central injury (Seijffers et al., 2007). Indeed, in transgenic mice in which ATF3 was over-expressed in DRG neurons, these mice exhibited enhanced regeneration comparable to that of the classical conditioning lesion. These data suggest that ATF3 coordinates the expression of a cohort of factors important for peripheral nerve regeneration.

The Filbin lab first discovered that cAMP was rapidly and robustly induced following a peripheral lesion (Qiu et al., 2002) and that cAMP could mimic the conditioning lesion effect. Subsequent studies (Gao et al., 2004) showed that cAMP's effects on regenerative axon growth required the downstream transcription factor CREB and that constitutively active CREB promoted regeneration after a dorsal column lesion.

The Zou lab showed (Zou et al., 2009), that following a conditioning lesion Smad1, a BMP-responsive transcription factor, gets phosphorylated and accumulates in the nucleus and is required for conditioning lesion-induced growth. And while knockdown of Smad1 *in vitro* impairs the axonal growth, intraganglionic injection of BMP2 or BMP4, promoted axonal growth and mimicked the effects of the conditioning lesion. The well-known tumor suppressor protein and transcription factor p53 has also been shown to be required for axonal regeneration *in vivo* in a model of facial nerve transection in which p53 null mice exhibited impaired regeneration (Tedeschi et al., 2009).

## Summary

For an axon to regenerate, the neuron will have to first survive the initial damaging insult be it a stroke, traumatic brain injury or the severing of a nerve. An injury response must be then transmitted back to the cell body. Next, a transcriptional program must be initiated as well as sustained activation of signaling pathways to coordinate both elaboration of growth cones at the distal axon tip as well as long distance growth of the axon shaft via organized transport of cytoskeletal and other essential proteins required for a growing axon. *A priori*, one would predict that in order for an axon to regrow over long distances that many factors would have to be concurrently and coordinately activated for such a complex process to be undertaken. As such, activation of factors more relatively upstream would be predicted to be more effective at driving regeneration (Blesch et al., 2012). That said it is perhaps shortsighted to think of signaling pathways as being strictly linear as there is most certainly crosstalk among different pathways. Along these lines the studies of Geschwind and colleagues (Michaelevski et al., 2010; Parikshak et al., 2015; Chandran et al., 2016), provide a path forward as to how to network-based, genomics approaches will inform the basic biology of neuronal regeneration.

The ultimate goal of the regenerative work described here is to improve outcomes for patients who have sustained neuronal injury. Recent work (Friedli et al., 2015) highlights the utility and relevance of non-human primate clinical spinal cord injury models. Interestingly, these studies revealed significant interspecies differences in the mechanisms of corticospinal tract regeneration during motor recovery when comparing rodents to primates and humans. Finally, combinatorial and holistic approaches that incorporate rehabilitation, pharmacology, neuromodulation (Moraud et al., 2016; Wenger et al., 2016) as well as emerging technologies like the exoskeleton (Gad et al., 2015; Miller et al., 2016) raise hopes that meaningful, functional recovery is achievable in the foreseeable future.

## Author contributions

The author confirms being the sole contributor of this work and approved it for publication.

## Funding

The author is funded by the Army Research Laboratory Collaborative Research Program and United States Military Academy Faculty Development Research Funds.

### Conflict of interest statement

The author declares that the research was conducted in the absence of any commercial or financial relationships that could be construed as a potential conflict of interest.
